# Effects of Core Disgust and Moral Disgust on Moral Judgment: An Event-Related Potential Study

**DOI:** 10.3389/fpsyg.2022.806784

**Published:** 2022-06-15

**Authors:** Dan Tao, Yue Leng, Jiamin Huo, Suhao Peng, Jing Xu, Huihua Deng

**Affiliations:** ^1^School of Biological Science and Medical Engineering, Southeast University, Nanjing, China; ^2^Research Center for Learning Science, Southeast University, Nanjing, China; ^3^Key Laboratory of Child Development and Learning Science of Ministry of Education, Southeast University, Nanjing, China; ^4^Department of Psychology, College of Literature, Science, and the Arts (LSA), University of Michigan, Ann Arbor, MI, United States

**Keywords:** moral judgment, core disgust, moral disgust, pure domain, event-related potentials (ERP)

## Abstract

Core disgust is elicited by physical or chemical stimuli, while moral disgust is evoked by abstract violations of moral norms. Although previous studies have pointed out these two types of disgust can affect behavior and spatial dimensions of moral judgment, less is known about how moral and core disgust affect the temporal neural processing of moral judgment. In addition, whether moral and core disgust are only related to purity-based moral judgment or all kinds of moral judgment is still controversial. This study aimed to explore how core and moral disgust affect the neural processing of purity-based moral judgment by using affective priming and moral judgment tasks. The behavioral results showed that the severity of moral violation of non-purity ones is higher than purity ones. The event-related potentials (ERP) results mainly revealed that earlier P2 and N2 components, which represent the automatic moral processes, can differentiate neutral and two types of disgust rather than differentiating moral domain, while the later N450, frontal, and parietal LPP components, which represent the conflict detection and, later, cognitive processing can differentiate the purity and non-purity ones rather than differentiating priming type. Moreover, core and moral disgust priming mainly differed in the purity-based moral processing indexed by parietal LPP. Our findings confirmed that the disgusting effect on moral judgments can be explained within the framework of dual-process and social intuitionist models, suggesting that emotions, including core and moral disgust, played an essential role in the automatic intuition process. The later parietal LPP results strongly supported that core disgust only affected the purity-based moral judgment, fitting the primary purity hypothesis well. We show how these theories can provide novel insights into the temporal mechanisms of moral judgment.

## Introduction

From an evolutionary perspective, disgust is an emotional system that has evolved to detect signs of pathogens, parasites, and toxins, and stimulate behavior to reduce the risk of acquiring them. Recently, it has been speculated that disgust may stem from repulsion to unpleasant foods (e.g., bitter substances), which helps keep us away from contamination and infection (Curtis, 2014; Kavaliers et al., 2019). In the evolution of human society, disgust is further divided into several subtypes, such as core disgust and moral disgust. Core disgust, also known as physical disgust, is caused by physical or chemical stimuli, such as feces and unhygienic individuals. Moral disgust is evoked by abstract violations of moral norms, including prostitution, bestiality, deception, and theft (Chapman and Anderson, 2013; Zhang et al., 2015).

It should be noted that some experimental studies have shown that core disgust and moral disgust are homogenous. For instance, both can cause activation of the facial levator muscle and exhibit oral rejection characteristics (Chapman et al., 2009). In addition, numerous functional magnetic resonance imaging (fMRI) studies have found that core and moral disgust share the same brain network composed of the basal ganglia, amygdala, thalamus, anterior cingulate gyrus (ACC), precuneus, anterior hippocampus, and insula (Chapman and Anderson, 2013). On the contrary, many research results also pointed out the heterogeneity of core disgust and moral disgust. Firstly, some fMRI studies claimed that core and moral types of disgust are associated with different brain regions. Core disgust can activate the left fusiform gyrus, lingual gyrus, and posterior cingulate cortex, while moral disgust can activate the temporoparietal junction (TPJ), temporal pole, superior temporal sulcus (STS), superior frontal lobe, and orbital prefrontal cortex (OFC) (Vicario et al., 2017). Secondly, several event-related potentials (ERP) studies proposed that core disgust and moral disgust stimuli can induce different ERP components. For example, Luo et al. (2013) explored the difference between core disgust and moral disgust through a lexical judgment task. They found that core disgust words strengthened the parietal EPN, frontal N320, and central late positive component (LPC) responses, while moral disgust enhanced the parietal N400 responses. Such results indicated that core disgust regulated word processing from the lexical evaluation stage to the post-semantic integration stage; in contrast, moral disgust did not affect the early stages of text processing and even showed signs of weakening in this implicit emotional task (Luo et al., 2013). By adopting an oddball paradigm, Zhang et al. (2015) found that core disgust images induced more positive deflection of the frontal N1 and P2, parietal P3, and central LPP, while moral disgust images induced more negative-going deflection of the N2 in the fronto-central region (Zhang et al., 2015), which is consistent with the conclusions of Luo et al.’s (2013) study that core disgust and moral disgust are processed through different cognitive neural mechanisms (Luo et al., 2013). Thirdly, several physiological studies provided evidence for the heterogeneity of core disgust and moral disgust. Ottaviani et al. (2013) reported that the core disgust group showed increased somatic aversion to parasympathetic nervous system activity and no synchronous changes in the heart rate, while the moral disgust group showed symptoms of reduced vagal tone and increased autonomic imbalance. In addition, some other studies also found that moral disgust increased the heart rate, while core disgust decreased the heart rate (Peng et al., 2013; Gilchrist et al., 2016).

In conclusion, several previous studies supported the homogeneity of core disgust and moral disgust by showing overlaps between them, while others highlighted the heterogeneity between them by providing convincing evidence. For example, common brain regions shared by two kinds of disgust may work differently. Specifically, both core and moral types of disgust activate the ACC, which involves heart rate management. However, studies have shown that the two types of disgust have opposite effects on the heart rate, which reduces the persuasiveness of homogeneity.

Moral judgment is the process of judging and inferring one’s own or others’ moral behaviors by using existing moral concepts (Decety et al., 2012). In recent years, numerous behavioral and neurological studies have demonstrated that emotions play an essential role in moral judgment (Huebner et al., 2009; McAuliffe, 2018). Such effects have been explained through the dual-process model (Paxton and Greene, 2010) and the social intuitionist model (Greene and Haidt, 2002), which claim that social-emotional components determine moral choice. As a basic emotion, disgust has received particular attention for its putative role in moral judgment (Landy and Goodwin, 2015). Researchers proposed that both unconscious and conscious types of disgust or disgust-induced stimulus activation can change moral judgments (Lim et al., 2017). Many theorists of morality have also considered how disgust might engage in moral judgment. Pizarro et al. (2011) proposed the amplification hypothesis, which emphasizes that the experience of disgust amplifies moral evaluations; it makes wrong things seem even more wrong (Pizarro et al., 2011). Some studies have demonstrated that individuals induced to feel disgusted can amplify the severity of moral condemnation (Landy and Goodwin, 2015; Lim et al., 2017). Other studies concerned with correlational results, which are consistent with a causal link between disgust and moral judgment. Some studies have shown that disgust sensitivity can positively predict the severity of moral judgments (Pizarro et al., 2011; Chapman and Anderson, 2013), especially concerning the punitive judgments of bodily and sexual purity (Horberg et al., 2009). These results above provide convincing evidence for the model that moral judgment is primarily driven by emotion. However, Olatunji and Puncochar (2016) reported that the participants’ judgments in the disgust condition did not differ from those of the participants in the neutral condition; no amplification effect of disgust induction was observed (Olatunji and Puncochar, 2016). A recent meta-analysis has also argued against this causality in moral judgment through many replicated behavioral experiments (Landy and Goodwin, 2015; Johnson et al., 2016), suggesting a need for more rigorous research on this topic.

From the behavioral studies above, it is still a question of whether the amplification effect exists. While recent imaging and neuropsychological studies have appeared to support that emotions can modulate the processes of moral judgments and play an essential role (Baez et al., 2017), until now, only one fMRI study initiated to investigate the neural mechanism underlying moral judgment by disgust priming and found that enhanced functional connectivity between the TPJ and dorsal medial prefrontal cortex (dmPFC), which indicated that disgust stimuli modulate moral judgment by altering the integration of moral reasoning and social information (Lim et al., 2017). However, imaging data are insufficient to determine whether the disgust priming effect is activated in which phase of moral cognition and whether the disgust priming effect occurs upstream, downstream, or even in parallel to moral processing (Huebner, 2013). The early and late ERP components can stand for different processes in the previous electrophysiological studies. A series of ERP studies have shown that moral judgment can induce the P2 and N2, which are closely related to early automatic processing, and the frontal and parietal LPPs are related to the later controlled and elaborative stages of moral processing (Yoder and Decety, 2014; Jiang et al., 2020). These components we discussed above represent cognitive reasoning and intuitive emotion processes in moral judgment (White et al., 2017; Yoder et al., 2021). Moreover, the N400-like component is related to conflict detection in an affective priming paradigm (Aguado et al., 2013). Therefore, using a high temporal-resolution ERP technique to investigate the temporal dynamics of neural processing of how disgust affects moral judgment might be a good approach.

Besides, although there is an emerging consensus that disgust may play a role in moral judgment, a more controversial proposition is whether this role is limited to general moral violations (e.g., vandalism and dropping litter), or whether disgust also affects a “pure” domain (e.g., sexual crimes and bestiality) (Hanah and Adam, 2014). Purity is concerned with avoiding pathogens through the regulation of sexual and eating behaviors (Crone et al., 2018). Previous behavioral studies showed that people tend to make more strictly moral judgments in the non-purity domain and judge the purity domain to be more moral (Landy and Goodwin, 2015; Olatunji et al., 2016). The imaging and electrophysiological studies also showed that the processing of purity-based moral judgment was different from that in other domains (Dungan and Young, 2019; Jiang et al., 2020). So far, numerous empirical studies have formed two competing hypotheses (Wagemans et al., 2018). One is the primarily binding hypothesis, which claims that disgust is automatically used to judge social events and can affect general moral judgment as a kind of physical reaction (Rozin et al., 2008). Wheatley and Haidt (2005) found moral judgments can be made more severely by giving a posthypnotic suggestion to feel disgusted (Wheatley and Haidt, 2005). Such findings are consistent with some literature, which indicated that disgust priming would strengthen individuals’ condemnation of various immoral incidents (Schnall et al., 2008; Danovitch and Bloom, 2009). Moretti and di Pellegrino (2010) also found that, compared to sadness and neutral conditions, disgust led to more rejection of unfair distribution (Moretti and di Pellegrino, 2010). On the contrary, the primary purity hypothesis claims that disgust is only related to purity-based moral judgment (Horberg et al., 2009, 2011). Horberg et al. (2009) found that both situational disgust and trait disgust can precisely predict people’s moral condemnation of purity norms. Helzer and Pizarro (2011) even found purity reminders caused people to condemn abnormal sexual behaviors severely, but the immoral behaviors unrelated to purity were not affected; purity reminder and disgust priming have similar effects, probably because both of them could induce intrinsic anxiety about disease infection (Helzer and Pizarro, 2011). Such controversial results may be due to the confusing classification of disgust priming stimuli (Luo et al., 2013). These results suggest that the role of disgust in moral judgment is not limited to social norms, and that disgust may play a role in the purity-based moral domain. Whether different kinds of disgust affect moral judgment and whether it affects all sorts of moral judgments (i.e., the perceived wrongness of specific purity behaviors and situations) remain unknown. Therefore, we classified disgust priming into moral disgust priming and core disgust priming, attempting to explore how the different kinds of disgust affect purity-based moral judgment.

As discussed above, less is known about how moral and core types of disgust affect the neural processing of purity-based moral judgment. This current ERP study attempted to compare the differences in the time course of brain responses of the purity-based and non-purity-based moral processing under different types of priming stimuli. The target stimuli were selected from the purity-related (e.g., sexual crimes) or non-purity-related moral domain (e.g., vandalism). The priming stimuli referred to core disgust (e.g., feces) and moral disgust (e.g., incest). Based on previous work (Landy and Goodwin, 2015; Olatunji et al., 2016; Jiang et al., 2020), we predicted that, in terms of behavior, disgust priming could not judge the immoral behaviors more harshly, but it may make judgments in the non-purity domain more strictly. Purity and non-purity moral processing would induce different ERP components, especially some later ERP components, such as the N450 and LPP, representing conflict detection and resolution (McCleery et al., 2011), might help distinguish them. Additionally, according to the previous studies and models (Lim et al., 2017), disgust can affect moral judgments; we hypothesized that, compared to neutral priming, both moral and core types of disgust priming can induce N2, P2, frontal LPP, and parietal LPP components in moral judgment.

## Materials and Methods

### Participants

Overall, 286 students (age range, 18–28 years) from Southeast University in China participated in this study and completed two questionnaires, including the Marlowe-Crown Social Desirability Scale (MCSD) (Crowne and Marlowe, 1960), for accessing social expectation effect and Three Domains of Disgust Scale (TDDS) for accessing disgust sensitivity (Olatunji et al., 2012). Data point that were greater than 2 SDs from the mean for each scale in the data set were excluded since we did not include the subjects with too high or too low sensitivity to social desirability and disgust. Finally, 100 participants were selected to participate in the EEG experiment, and they were randomly divided into three groups. Twenty-four participants were excluded from the analysis due to excessive movement artifacts in their EEG recordings or declined to participate in the EEG portion of the assessment. Thus, EEG data of 76 participants (34 females, age = 21.71 ± 20 years) were analyzed, 26 in the moral disgust priming group, 24 in the core disgust priming group, and 26 in the neutral group. The ANOVA results showed that there was no significant difference in social expectation and disgust sensitivity among these three groups [*F*(2,73) = 1.12, *p* > 0.1; *F*(2,73) = 0.44, *p* > 0.1].

All the participants were right-handed, had no history of neurological problems, and had normal or corrected-to-normal vision and hearing. After the experiment, each of them received a compensation of 100 RMB. All the participants signed written informed consent, and this study was conducted following the regulations of the Ethics Committee of Affiliated Zhongda Hospital, Southeast University, China.

### Materials

#### Affective Priming Pictures

Three types of priming pictures were selected from disgust-related-images (DIRTI) database (Haberkamp et al., 2017). The DIRTI was initially validated with over 240 disgust pictures and 60 matched neutral pictures from 200 participants, with normative ratings currently available for all images on disgust, fear, valence, and arousal (the arousal, disgust, and fear scales ranged from 0 = *none* to 100 = *extremely strong*). After balancing the other dimensions (fear, valence, and arousal) to ensure no significant difference among the three types of pictures on each dimension, then, 36 pictures were obtained, including 12 moral disgust pictures, 12 core disgust pictures, and 12 neutral pictures. One-way ANOVA on the disgust level was conducted. The results showed that there were significant differences among three types of pictures, *F*(1,46) = 2,481.15, *p* < 0.001, the *post hoc* test showed the disgust level for core disgust pictures (74.06 ± 7.52) was higher than neutral pictures (11.25 ± 2.05) (*p* < 0.05), and the disgust level for moral disgust pictures (72.85 ± 10.35) was higher than neutral pictures (11.25 ± 2.05) (*p* < 0.05), but there was no difference in the disgust level between the core disgust pictures and the moral disgust pictures (*p* > 0.1).

#### Moral Judgment Pictures

A total of 66 pictures (33 purity immoral, 33 non-purity immoral) were selected from the socio-moral image database (Crone et al., 2018); each picture was rated by six experts who did not take part in the EEG study, aiming at validating the moral content, valence, purity, and arousal of the selected stimuli. The pictures were rated on a scale from 1 (i.e., low) to 5 (i.e., high) on four dimensions, namely, moral content, valence, purity, and arousal. The *t*-test for all dimensions of the two sets of pictures was conducted. The results showed the ratings for purity and non-purity pictures differed significantly in purity uniqueness (*t* = −16.07, *df* = 47.11, *p* < 0.001), but there were no significant differences in arousal (*t* = 1.58, *df* = 55.58, *p* > 0.1), valence (*t* = −0.86, *df* = 54.02, *p* > 0.1), and moral content (*t* = −0.96, *df* = 64, *p* > 0.1).

### Design and Procedures

**FIGURE 1:**
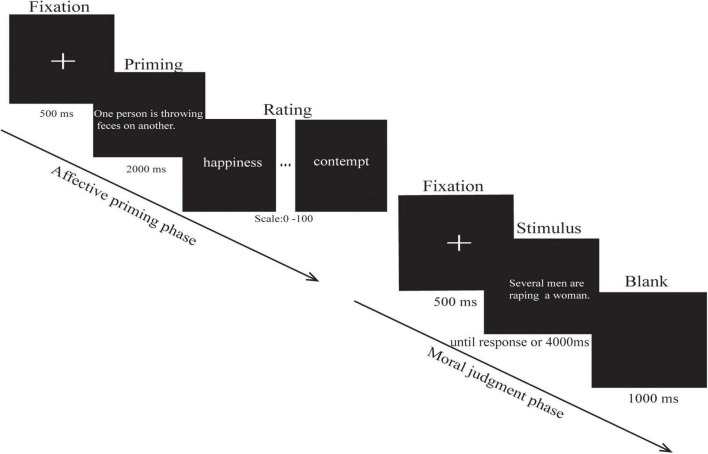
Schematic representation of a single affective priming trial and a single moral judgment trial. The formal experiment consisted of three blocks; each block contained an affective priming phase and a moral judgment phase. (The original version of the images cannot be provided for copyright reasons, so we added a short textual description of what the image represented.) (Crone et al., 2018).

The experimental procedure was programmed using E-prime software (Version 2.0, Psychology Software Tools, Inc.). The stimulus was presented on a cathode ray tube monitor at a 60 Hz refresh rate. The distance between the subject and the screen was about 60 cm, and the visual angle was about 3.8° × 4.9°. The 100 participants responded by keystrokes on the keyboard. In addition, 34 participants were assigned to the moral disgust priming task, 33 participants were assigned to the core disgust priming task, and 33 participants were assigned to the neutral priming task. The task procedure is shown in Figure 1.

The experimental session consisted of 24 trials in the affective priming phase and 132 trials in the moral judgment phase, including 12 priming pictures and 66 moral judgment pictures, each of which was presented two times. To ensure the priming effect is time-efficient, two phases alternated with each other. The entire experiment comprised three blocks of two phases each, and the two phases included the affective priming phase and the moral judgment phase. In the affective priming phase, there are eight trials in each block; each trial began with a fixation point presented for 500 ms, followed by the affective priming pictures, and each picture was automatically presented for 2,000 ms, and then the participants were asked to fill out the visual analog scales (VAS) (Klimek et al., 2017) by rating the degree of six emotions (happiness, anxiety, disgust, anger, sadness, and contempt; 0 = *no feeling at all*, 100 = *extremely strong*) for each picture. In addition, three groups of the participants rated moral disgust, core disgust, and neutral pictures separately. Upon VAS completion, the participants pressed the space bar to get into the moral judgment task and were reminded to restrict their movements to avoid interference with the EEG recording. Each block had 44 trials for the “purity and non-purity” in the moral judgment phase. Each trial began with a fixation point presented for 500 ms, followed by the stimulus, and the stimulus disappeared once the participant responded, and if no response was given within 4,000 ms, the next trial was presented. The inter-trial interval was a 1,000-ms blank screen. The participants were asked to rate how morally wrong they found each transgression on a scale from 0 (*not morally wrong at all*) to 8 (*extremely morally wrong*). The order of blocks was counterbalanced among the participants. The pictures in each block were randomly selected from the full set of picture stimuli, and the sequence of trials in each block was pseudo-random. The program automatically recorded both reaction results and reaction time, and the EEG data were recorded in the moral judgment phase.

### EEG Recording and Analysis

According to the International 10–20 system, EEG was recorded from 64 scalp sites using tin electrodes installed in elastic caps (NeuroScan Inc., Herndon, VA, United States). The reference electrodes were placed on the left and right mastoids. All electrode impedance was maintained below 5 kΩ. All the signals were sampled at 500 Hz, and bandpass filtering was performed in the frequency range of 0.1–100 Hz. At the same time, by placing electrooculogram (EOG) electrodes on the outside of the upper left eyebrow and the lower eyebrow, the horizontal electrooculogram (HEOG) and vertical electrooculogram (VEOG) were recorded.

Neuroscan 4.5 was adopted to perform offline analysis of EEG data recorded in the formal experiments. EEG data were low-pass filtered below 30 Hz (24 dB/oct). The eye movement correction algorithm combined regression analysis with the artifact average to correct ocular artifacts (Semlitsch et al., 1986). All trials with EEG voltages exceeding the ±80-μV threshold at any electrode during recording were excluded from further analysis. At the start of each stimulus, a time lock was extracted from successive EEG recordings, including a pre-stimulation baseline of 200 ms, with duration of 1,000 ms.

According to previous studies (Larson et al., 2014; Hill et al., 2019) and the visual inspection of grand-averaged waveforms and scalp topographies in the present study, the P2, N2, N450, frontal LPP, and the parietal LPP (analyzed separately at early and middle latency ranges) were analyzed. The peak amplitudes of the P2 in the time window of 150–200 ms, N2 in the time window of 190–350 ms, and the mean amplitudes of the N450 in the time window of 400–600 ms and frontal LPP in the time window of 700–900 ms were extracted at electrodes at the frontal and frontal-central areas (i.e., F3, Fz, F4, FC3, FCz, and FC4). The mean amplitude of the LPP at parietal and parietal-central areas (i.e., CP3, CPZ, CP4, P3, PZ, and P4) was computed in the time window of 400–600 ms.

For all ERP components, the data were cast into a mix-factor repeated-measures ANOVA using statistical product and service solutions (SPSS 18.0; SPSS, Inc., Chicago, IL, United States), with image type (purity, non-purity) and hemisphere (left, right) as within-subject factors, priming type (moral disgust, core disgust, and neutral) as a between-subject factor. For all statistical analyses, the significance level was set at 0.05. The Greenhouse–Geisser correction for non-sphericity was applied whenever appropriate. The Bonferroni correction was used for multiple comparisons. Significant interactions were analyzed using simple-effects models. Effect sizes were presented as partial eta-squared ($\eta_{p}^{2}$).

## Results

### Subjective-Rating Data

A one-way ANOVA (priming type: moral disgust, core disgust, and neutral) was conducted separately for the ratings of six emotions. The Bonferroni correction was used for multiple comparisons. As shown in Table 1, the results showed significant differences in happiness, *F*(2,73) = 82.02, *p* < 0.001, η^2^ = 0.69; anxiety, *F*(2,73) = 25.67, *p* < 0.001, η^2^ = 0.41; disgust, *F*(2,73) = 426.04, *p* < 0.001, η^2^ = 0.92, anger, *F*(2,73) = 130.78, *p* < 0.001, η^2^ = 0.78, sadness, *F*(2,73) = 111.75, *p* < 0.001, η^2^ = 0.75), and contempt, *F*(2,73) = 32.50, *p* < 0.001, η^2^ = 0.47. *Post hoc* tests showed that higher scores for the neutral priming group than for the moral disgust priming group (*p* < 0.001) and higher scores for the neutral priming group than for the core disgust priming group (*p* < 0.001) in happiness; higher scores for the moral disgust priming group than for the neutral priming group (*p* < 0.001) and higher scores for the core disgust priming group than for the neutral priming group (*p* < 0.001) in anxiety; higher scores for the moral disgust priming group than for the neutral priming group (*p* < 0.001) and higher scores for the core disgust priming group than for the neutral priming group (*p* < 0.001) in disgust; higher scores for the moral disgust priming group than for the core disgust priming group (*p* < 0.001) and higher scores for the core disgust priming group than for the neutral priming group (*p* < 0.001) in anger; higher scores for the moral disgust priming group than for the core disgust priming group (*p* < 0.001) and higher scores for the moral disgust priming group than for the neutral priming group (*p* < 0.001) in sadness; higher scores for the moral disgust priming group than for the core disgust priming group (*p* < 0.001) and higher scores for the core disgust priming group than for the neutral priming group (*p* < 0.001) in contempt.

**TABLE 1:** The means and SD of the variables separated by affective manipulation.

Emotion	Moral disgust		Core disgust		Neutral	
	*M*	SD	*M*	SD	*M*	SD
Happiness	9.11	12.21	3.68	7.89	55.60	23.11
Anxiety	41.56	20.75	40.42	26.33	6.70	8.83
Disgust	81.77	11.77	87.24	14.62	4.13	6.62
Anger	75.37	16.62	35.35	24.33	1.32	2.98
Sadness	54.96	18.20	9.17	14.24	2.96	5.03
Contempt	48.20	22.54	27.01	28.71	1.42	3.87

### Behavior Data

**FIGURE 2:**
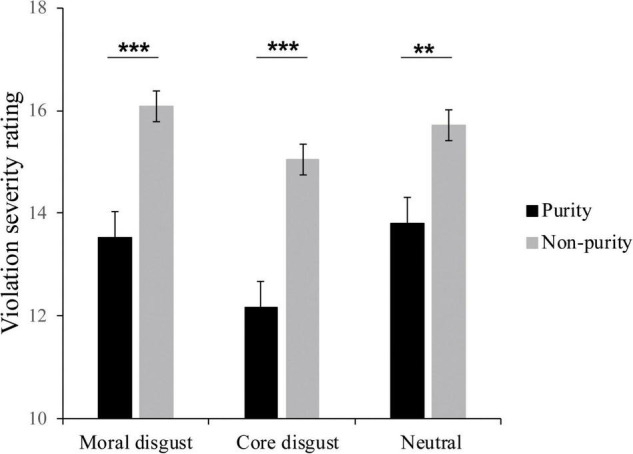
The violation severity ratings of behavior response for each condition in moral disgust, core disgust, and neutral groups; the means and standard errors are shown, *p < 0.05, **p < 0.01, ***p < 0.001.

Repeated measure ANOVA was performed for the violation severity ratings, with image type (purity and non-purity) as within-subject factors and priming type (moral disgust, core disgust, and neutral groups) as a between-subject factor. The Bonferroni correction was used for multiple comparisons. As seen in Figure 2, the results showed the main effect of image type, *F*(1,73) = 99.18, *p* < 0.001, $\eta_{p}^{2}=0.58$, the participants rated the non-purity pictures (15.65 ± 0.29), higher on moral violation severity than the purity pictures (13.17 ± 0.36). Besides, neither other main effect nor interaction effect was significant, *ps* > 0.1.

### Event-Related Potentials Data

**FIGURE 3:**
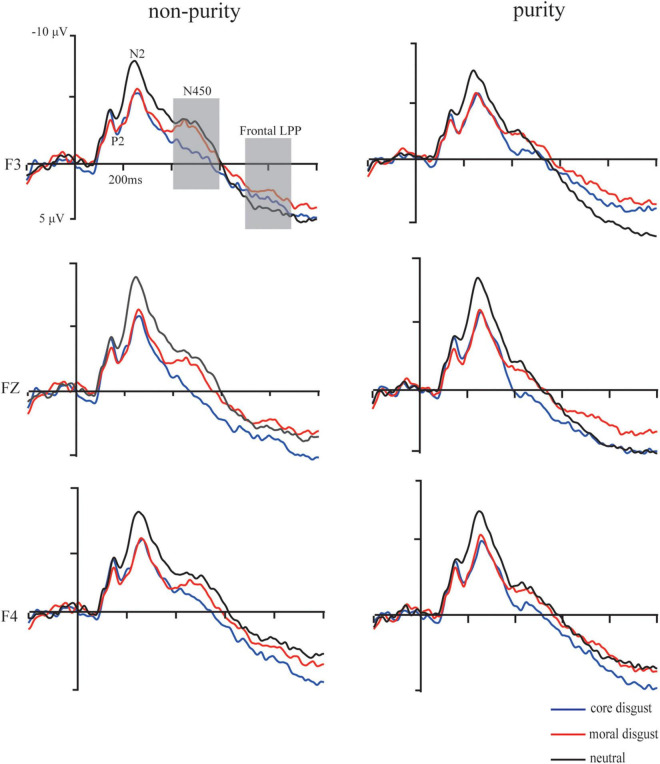
Grand-average event-related potential waveforms for moral disgust (a red solid line), core disgust (a blue solid line), and neutral (a black solid line) groups under purity conditions at F3, Fz, and F4 electrodes. The gray bars highlight the time window of the N450 (400–600 ms) and frontal LPP (700–900 ms).

**FIGURE 4:**
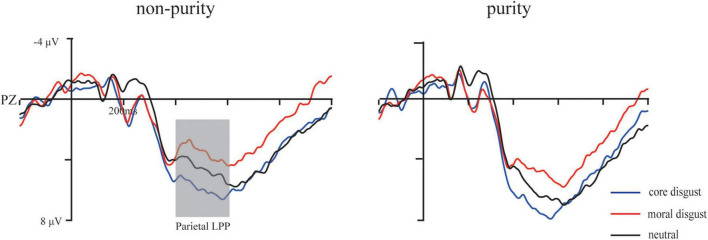
Grand average waveforms for moral disgust, core disgust, and neutral groups under purity conditions at F3, Fz, and F4 electrodes. The gray bar highlights the time window of the parietal LPP (400–600 ms).

Grand average ERP waveforms showing the significant difference among three priming types are illustrated in Figures 3, 4, respectively.

**The P2 (150–200 ms):** For the peak amplitude, the ANOVA showed a significant main effect of priming type, *F*(2,73) = 4.61, *p* < 0.05, $\eta_{p}^{2}=0.11$, the *post hoc* test showed that with ERP responses being more positive going in the moral disgust priming group than those in the neutral priming group (*p* < 0.05) and more positive going in the core disgust priming group than those in the neutral priming group (*p* < 0.05). Besides, neither other main effect nor interactions reached significant, *ps* > 0.1. For the latency, neither the main effect nor the interactions reached significant, *ps* > 0.1.

**The N2 (190–350 ms)**: For the peak amplitude, the ANOVA showed a significant main effect of priming type, *F*(2,73) = 3.91, *p* < 0.05, $\eta_{p}^{2}=0.08$, the *post hoc* test showed that, with ERP responses being more negative going, amplitude in the neutral priming group than those in the moral disgust priming group (*p* < 0.05) and more negative going in the neutral priming group than those in the core disgust priming group (*p* < 0.05). Besides, neither other main effects nor the interactions reached significant, *ps* > 0.1. For the latency, neither the main effect nor the interactions reached significant, *ps* > 0.1.

**The N450 (400–600 ms):** For the mean amplitude, ANOVA showed a significant main effect of image type, *F*(1,73) = 14.64, *p* < 0.001, $\eta_{p}^{2}=0.18$, with ERP responses being more negative going for non-purity trials than for purity trials. Moreover, a three-way interaction among image type, priming type, and hemisphere was significant, *F*(2,146) = 3.35, *p* < 0.05, $\eta_{p}^{2}=0.08$. Furthermore, simple effect analysis indicated more negative-going amplitude for non-purity trials than those for purity trials over the left hemisphere in the moral disgust group, *p* < 0.05, but not over the right hemisphere, *p* > 0.1. Besides, neither other main effects nor the interactions reached significant *ps* > 0.1.

**The frontal LPP (700–900 ms):** For the mean amplitude, ANOVA results showed a significant main effect of image type, *F*(1,73) = 6.99, *p* < 0.05, $\eta_{p}^{2}=0.87$, with ERP responses being more positive going for purity trials than for non-purity trials. Additionally, there was a significant interaction between hemisphere and image type, *F*(2,146) = 9.94, *p* < 0.001, $\eta_{p}^{2}=0.12$. Follow-up simple effect analysis indicated more positive-going amplitude for purity trials than those for non-purity trials over the left hemisphere, *p* < 0.01, but not over the right hemisphere, *p* > 0.1. Besides, neither other main effects nor the interactions reached significant *ps* > 0.1.

**The parietal LPP (400–600 ms):** For the mean amplitude, ANOVA showed a significant main effect of image type, *F*(1,73) = 35.1, *p* < 0.001, $\eta_{p}^{2}=0.33$, with ERP responses being more positive going for purity trials than for non-purity trials. Additionally, an interaction between image type and priming type, *F*(2,146) = 2.78, *p* < 0.05, $\eta_{p}^{2}=0.07$. Furthermore, simple effect analysis indicated more positive-going amplitude in the core disgust group than those in the moral disgust group for the purity domain, *p* < 0.05, but not for the non-purity domain, *p* > 0.1. Besides, neither other main effects nor the interactions reached significant *ps* > 0.1.

## Discussion

The current study adopted the affective priming and the moral judgment paradigm to explore the temporal dynamic effects of moral disgust, core disgust, or neutral image exposure on moral judgment. As expected, the behavior results showed that the participants rated strict violations as more morally wrong for non-purity ones than purity ones regardless of priming type. This finding is consistent with previous studies that pointed out disgust is unrelated to moral judgments about purity or other domains, and people usually make stronger moral condemnation of behaviors for violations of the non-purity domain and consider the purity domain as more moral (Horberg et al., 2009; Olatunji et al., 2016). Our ERP results are the first to provide convincing evidence that the earlier P2 and N2 components, indexing the automatic conflict processes can distinguish between neutral and two types of disgust but cannot distinguish the moral domain. Still, the later N450, frontal, and parietal LPP components can differentiate the purity and non-purity ones but cannot differentiate priming type. In addition, the parietal LPP indexing the cognitive control processes can differentiate core disgust and moral disgust in the purity but not in the non-purity domain.

### Both Moral and Core Types of Disgust Priming Affect Automatic Processing in Moral Judgment

The early frontal P2 was only sensitive to priming type, with more positive amplitude in moral disgust and core disgust priming groups than that in the neutral group. We believe that this is early emergence of the main effect of the stimuli that occur automatically. Previous studies have suggested that P2 reflects the early automatic attention and conflict detection processing (Chen et al., 2009) or emotional arousal of perceived moral stimuli (Chen et al., 2009; Sarlo et al., 2012). Such a P2 effect was also observed in other ERP experiments. In the time window of 140–210 ms, the P2 amplitude of the core disgust priming group was larger than that of the neutral priming group, indicating that the core disgust-evoking pictures were automatically processed (Zhang et al., 2015). Thus, in this study, both the moral disgust group and the core disgust group can affect the early automatic stage of moral judgment, resulting in an enhanced P2 than the neutral group. Specifically, the P2 effect can indicate the early influence of disgust priming on moral judgment at an early stage, and disgust deepens automatic processing.

In the present study, the subsequent N2 amplitude (early frontal negativity peaking at 250 ms) was also modulated by the priming type, and the negative amplitude of the neutral priming group was larger than that of the moral disgust group and the core disgust group. Pletti et al. (2019) reported that the frontal N2 component was related to cognitive conflict (Pletti et al., 2019), detection of novelty (Harsay et al., 2012; Yoder and Decety, 2014), and violation of expectations (Folstein and Van Petten, 2008). Some studies on moral judgment showed that the amplitude of N2 for moral behaviors was greater than for immoral behaviors and sensitive to moral valence and arousal, since immoral behaviors that violated social norms should be unexpected (Yoder and Decety, 2014; Gui et al., 2015; Lu et al., 2019). In our experiment, the amplitude of N2 was sensitive to disgust priming and maybe because moral and core disgust priming stimuli violated the participants’ expectations, leading to stronger conflicts in moral judgment and the emergence of conflict adaptation effects (Larson et al., 2014). Yang et al. (2019) also pointed out that defensive motivation can increase conflict adaptation (N2), which can be separated from more comprehensive changes in information processing (Yang et al., 2019), and disgust priming may produce defensive motivation. Thus, an enhanced disgust priming effect was associated with a lower N2 amplitude in moral judgment.

In brief, the current study provides evidence that disgust modulates the early automated processing in moral judgment, and disgust deepens automatic processing. Based on these important findings, the dual-process model (Paxton and Greene, 2010) and the social intuitionist model (Greene and Haidt, 2002) can support these well, which suggests that emotions play a critical role in the automatic intuition process. Both core and moral disgust effects are also equally applicable to the theory.

### Purity and Non-purity Moral Judgment Differed in Later Attentional Reallocation and Controlled Cognitive Processing

In addition to the P2 and N2, the later ERP components were influenced by image type rather than priming type. The N450 had a fronto-central scalp distribution similar to N2 in this study, and the negative amplitude of non-purity trials was larger than that of purity trials. This component was located at the anterior cingulate cortex (ACC) and widely existed in cognitive control tasks, such as the Stroop and Flanker. It may represent conflict detection, and it was more negative, following incongruent trials than following congruent trials (Larson et al., 2014; Pires et al., 2014). Previous studies proposed an enhanced N450 amplitude in conflict monitoring with increased incongruity (Chouiter et al., 2014). When the conflict was high, the increase in N450 amplitude was accompanied by an increase in inconsistent conflict monitoring. Other studies have also found that the N450 was sensitive to stimulating conflicts rather than responding to conflicts (Appelbaum et al., 2009). Therefore, the N450 difference between non-purity and purity in the present study can reflect the incongruence in the moral domain. Besides, the N450 showed a left-lateralized conflict effect only in the moral disgust priming group, which was consistent with similar previous findings in the typical negative priming task, and the N450 amplitude was greater in negative priming than in non-primed incongruent trials, which suggested this N450 effect can reflect interference detection (Hanslmayr et al., 2008). Some researchers also interpreted the N450 as an index of conscious inhibition processes (Pires et al., 2014) or an increased interference effect (Westa and Alainb, 2000), which may help overcome interference in the later stages (Tays et al., 2008). Similarly, the participants devoted more cognitive resources to inhibit the conflict of non-purity pictures, leading to more enhanced N450 after experiencing moral disgust priming in our study.

The subsequent frontal LPP component (starting at approximately ∼600 ms) showed greater activation for purity trials than non-purity trials only at the left hemisphere. Such a frontal component or an analogous component has been reported to be associated with top-down regulation, controlled cognition (Sabatinelli et al., 2007; Solomon et al., 2012), and attentional reallocation in response to motivationally salient stimuli (Dennis and Hajcak, 2009). Previous studies on social cognition and emotion suggested that frontal ERP components in the time window of 300–800 ms were associated with regulatory control of behavioral stereotypes (Amodio et al., 2014) and the resolution of conflicts between perspective choices (McCleery et al., 2011). Moreover, other works have found that local motivational attentional processes were more lateral to the left hemisphere than the right hemisphere (Gable and Harmon-Jones, 2010). Besides, ERP studies on moral judgment have proposed that the LPP at left frontal sites was associated with a later controlled cognitive reappraisal of helpful versus harmful behaviors (Yoder and Decety, 2014; Cowell and Decety, 2015). Therefore, the frontal LPP effect in our study suggested that purity stimuli need more attentional reallocation resources.

The parietal LPP also showed greater activation for purity trials than for non-purity trials. Numerous ERP studies on moral judgment have shown that late parietal positive deflection starting around 400 ms after the stimulus onset was associated with moral valence (Lu et al., 2019), moral arousal (Yoder and Decety, 2014; Cowell and Decety, 2015; Pletti et al., 2019), and moral content (Gui et al., 2015). It has been generally suggested that the parietal LPP reflects cognitive reappraisal of stimuli and attention redistribution in response to motivationally salient stimuli (Sabatinelli et al., 2007; Solomon et al., 2012). Specifically, such a component was even observed in the affective priming paradigm, the parietal LPP was sensitive to evaluative congruency, and the N400 reflected the semantic effects rather than evaluative congruency (Aguado et al., 2013). Analogously, it was consistent with our study that the N450 detected the incongruence of the moral domain, and the parietal LPP was enhanced by purity pictures than by non-purity pictures. These findings suggested that the evaluative congruency can modulate the LPP in the disgust priming paradigm, and the LPP was sensitive to the moral domain.

To sum up, the present study results have shown the N450, frontal LPP, and parietal LPP, representing different stages of moral processing, were sensitive to the moral domain in the priming paradigm. As discussed above, considerable evidence connected these components with incongruence detection, attentional reallocation, and evaluative congruency. The new evidence from our study revealed a complex pattern of moral disgust priming and congruency effects on the N450 component. This evidence suggested that congruency effects on the moral disgust priming were probably more complicated than in core disgust or neutral priming. In addition, both the frontal and parietal LPP components responded to the stimulus of the moral domain with more enhanced amplitude for purity pictures, suggesting that purity pictures attracted more attention and controlled cognitive reappraisal from individuals in moral judgment.

### Core Disgust Priming Affects Purity-Based Moral Judgment

It is worth noting that the parietal LPP amplitude was larger in core disgust priming than in moral disgust priming for purity, but this pattern did not exist for non-purity. Consistent with previous findings (Wagemans et al., 2018, 2019), disgust was associated with purity-based moral judgments, and both subliminal and conscious priming with core disgust stimuli or disgust-inducing stimuli can cause changes in moral judgments (Schnall et al., 2008; Ong et al., 2014). Notably, Luo et al. (2013) first reported a larger LPC (400–600 ms) for core-disgust words than for moral-disgust words (Luo et al., 2013), which may reflect further processes, such as attentional capture, emotional evaluation and further memory encoding, which are particularly sensitive to core disgust. No LPC effect was observed for the moral disgust words. Zhang et al. (2015) also found that core disgust pictures induced more positive deflection in the frontal N1 and P2, parietal P3, and central LPP, while moral disgust images only induced more negative deflection in the N2 in the fronto-central region (Zhang et al., 2015). In our experiment, similar neural responses reflected by the parietal LPP extended the prior finding, indicating that, after experiencing core disgust priming, the individuals devoted more mental resources and emotional evaluation to process purity-based moral judgment. The LPP in the time window of 400–700 ms was considered to represent the affective or motivational value of the stimuli in affective priming studies (Zhang et al., 2010; Eder et al., 2012). Therefore, the LPP effect in the current study suggested that more significant core-priming effects on the parietal LPP enhanced later cognitive processing or memory encoding. This result fits the primary purity hypothesis well; core disgust only affects the late-stage process in purity-based moral judgment. However, moral disgust priming does not apply to this hypothesis.

## Conclusion

The present study demonstrates that disgust priming cannot affect moral judgment, and people usually make stronger moral condemnation of behaviors for violations of the non-purity domain and moralize the purity domain on the behavior performance. Moral disgust and core disgust modulate purity-based and non-purity-based moral processing at different processing stages. Specifically, the ERP results suggested that moral and core disgust priming affect automatic moral judgment processing (indexed by the N2 and P2), and purity-based moral judgment needs more attentional reallocation and a controlled cognitive resource (indexed by the N450, frontal and parietal LPPs). After experiencing core disgust priming, the individuals devoted more mental resources and emotional evaluation to processing purity-based moral judgment, and a more significant core-priming effect was found on purity-based moral judgment (indexed by the parietal LPP). Our findings provide robust evidence on the dual-process and social intuitionist models, and it also applies to the primary purity hypothesis.

## Data Availability Statement

The original contributions presented in the study are included in the article/supplementary material, further inquiries can be directed to the corresponding author.

## Ethics Statement

The studies involving human participants were reviewed and approved by the Ethics Committee of Affiliated Zhongda Hospital, Southeast University, China. The patients/participants provided their written informed consent to participate in this study.

## Author Contributions

DT and JH designed the experiments. JH and SP carried out the experiments. DT and YL analyzed the experimental results and wrote the manuscript. JX, YL, and HD co-designed the experiment, advised on many aspects of the research, and co-wrote the text. All authors contributed to the article and approved the submitted version.

## Conflict of Interest

The authors declare that the research was conducted in the absence of any commercial or financial relationships that could be construed as a potential conflict of interest.

## Publisher’s Note

All claims expressed in this article are solely those of the authors and do not necessarily represent those of their affiliated organizations, or those of the publisher, the editors and the reviewers. Any product that may be evaluated in this article, or claim that may be made by its manufacturer, is not guaranteed or endorsed by the publisher.
